# The clinical response to infliximab in rheumatoid arthritis is in part dependent on pretreatment tumour necrosis factor α expression in the synovium

**DOI:** 10.1136/ard.2007.080440

**Published:** 2007-11-29

**Authors:** C A Wijbrandts, M G W Dijkgraaf, M C Kraan, M Vinkenoog, T J Smeets, H Dinant, K Vos, W F Lems, G J Wolbink, D Sijpkens, B A C Dijkmans, P P Tak

**Affiliations:** 1 Division of Clinical Immunology and Rheumatology, Academic Medical Center/University of Amsterdam, Amsterdam, The Netherlands; 2 Department of Clinical Epidemiology, Biostatistics and Bioinformatics, Academic Medical Center/University of Amsterdam, Amsterdam, The Netherlands; 3 Jan van Breemen Institute, Amsterdam, The Netherlands; 4 Department of Rheumatology, Slotervaart Hospital, Amsterdam, The Netherlands; 5 VU Medical Center, Amsterdam, The Netherlands

## Abstract

**Objective:**

To determine whether the heterogeneous clinical response to tumour necrosis factor (TNF)α blocking therapy in rheumatoid arthritis (RA) can be predicted by TNFα expression in the synovium before initiation of treatment.

**Methods:**

Prior to initiation of infliximab treatment, arthroscopic synovial tissue biopsies were obtained from 143 patients with active RA. At week 16, clinical response was evaluated using the 28-joint Disease Activity Score (DAS28). Immunohistochemistry was used to analyse the cell infiltrate as well as the expression of various cytokines, adhesion molecules and growth factors. Stained sections were evaluated by digital image analysis. Student t tests were used to compare responders (decrease in DAS28 ⩾1.2) with non-responders (decrease in DAS28 <1.2) and multivariable regression was used to identify the independent predictors of clinical response.

**Results:**

Synovial tissue analysis confirmed our hypothesis that the baseline level of TNFα expression is a significant predictor of response to TNFα blocking therapy. TNFα expression in the intimal lining layer and synovial sublining were significantly higher in responders than in non-responders (p = 0.047 and p = 0.008, respectively). The numbers of macrophages, macrophage subsets and T cells (all able to produce TNFα) were also significantly higher in responders than in non-responders. The expression of interleukin (IL)1β, IL6, IL18, IL10, E-selectin, intercellular adhesion molecule (ICAM)-1, vascular cell adhesion molecule (VCAM)-1, vascular endothelial growth factor (VEGF) and basic fibroblast growth factor (bFGF) was not associated with response to anti-TNFα treatment.

**Conclusion:**

The effects of TNFα blockade are in part dependent on synovial TNFα expression and infiltration by TNFα producing inflammatory cells. Clinical response cannot be predicted completely, indicating involvement of other as yet unknown mechanisms.

Tumour necrosis factor α (TNFα) blocking agents as treatment for rheumatoid arthritis (RA) were developed based on evidence that the pro-inflammatory cytokine TNFα plays an important role in the pathogenesis.^1^ Some patients however do not clinically respond to TNFα blockade. At present no factors have been identified that fully explain or predict the differential response.

One explanation for the heterogeneous clinical response may be found in the baseline variability in TNFα expression among individual patients.^2^ ^3^ Genetic studies have suggested that individuals predisposed to high TNF production could show worse responses to anti-TNFα therapy.^4^ ^5^ By contrast, a recent study using an in vitro bioassay suggested that good responsiveness to anti-TNF therapy is associated with significantly higher TNFα bioactivity at baseline compared to non-responding patients.^6^ Taken together, it remains to be determined which baseline cytokine profile distinguishes responding from non-responding patients in vivo. Another explanation for the diversity in response may be that inflammatory mediators other than TNFα drive different pathogenetic subsets of RA.

We hypothesised that the pretreatment TNFα level in the synovium might be related to clinical efficacy, where TNFα blocking therapy could be most effective in patients with high pretreatment TNFα levels, as previously suggested in a small pilot study.^7^ In a prospective study we obtained arthroscopic synovial tissue samples from 143 patients with RA prior to initiation of infliximab therapy. We examined the cell infiltrate as well as the expression of cytokines, adhesion molecules and growth factors to identify predictors of clinical response.

## PATIENTS AND METHODS

### Patients

Consecutive patients with RA according to the American College of Rheumatology (ACR) criteria were enrolled in the study. All failed at least two disease-modifying antirheumatic drugs (DMARDs) including methotrexate (MTX) and had a 28-joint Disease Activity Score (DAS28) of ⩾3.2 when included in the study. Patients were on stable maximal tolerable MTX treatment (5–30 mg/week). Oral corticosteroids (⩽10 mg/day) and non-steroidal anti-inflammatory drug (NSAIDs) were allowed if stable for at least 1 month prior to baseline. Concomitant medication was kept stable throughout the study. Previous use of a TNF blocking agent was an exclusion criterion. The Medical Ethics Committee of the Academic Medical Center, University of Amsterdam approved the protocol. All patients gave written informed consent.

### Treatment and evaluation of clinical response

All patients were treated with infliximab according to the label for RA in a dosage of 3 mg/kg intravenously at baseline, week 2, week 6 and subsequently every 8 weeks. The DAS28 was evaluated at baseline and weeks 4, 8, 12 and 16 by specially trained research nurses.

For the analysis the absolute change in DAS28 (ΔDAS28) at week 16 was dichotomised and defined as non-response (ΔDAS28 <1.2) vs response (ΔDAS28 ⩾1.2). The dichotomy of the ΔDAS28 (on average comparable with a 20% improvement in DAS28) was chosen because it is applied in daily clinical practice and required for prolongation of reimbursement for TNFα blocking therapy by insurance companies in The Netherlands. Response was evaluated at 16 weeks because a significant improvement is expected to occur within 3 to 4 months, after which alternative treatment should be considered.^8^

### Arthroscopy and synovial biopsy

Before the first infliximab infusion patients underwent a mini-arthroscopy under local anaesthesia to obtain synovial tissue samples from an actively inflamed knee, ankle, wrist or metacarpophalangeal joint.^9^ Biopsies were taken with 2 mm forceps (Storz, Tuttlingen, Germany) from six or more sites within the joint to minimise sampling error. Biopsies were immediately snap frozen en bloc in Tissue Tek OCT (Miles, Elkhart, Indiana, USA) after collection. Sections of 5 μm were cut in a cryostat and mounted on Star Frost adhesive glass slides (Knittelgläser, Braunschweig, Germany). Slides were stored at −80°C until immunohistochemical staining.

### Immunohistochemical analysis

Synovial sections were stained using the following monoclonal antibodies to analyse the infiltrate: anti-CD55 (67:Serotec, Oxford, UK) to detect fibroblast-like synoviocytes (FLS), anti-CD68 (EBM11: DAKO, Glostrup, Denmark) to detect macrophages, anti-CD3 (SK7, Becton Dickinson (BD), California, USA) for T cells, anti-CD22 (CLB-B-ly/1,6B11, The Netherlands) for B cells and anti-CD38 (HB7, BD) for plasma cells. CD163 (Ber-MAC3; DAKO) was stained to detect resident tissue macrophages; infiltrating macrophages were evaluated by detection of myeloid related protein (MRP)8 (8-5c2, BMA Biomedicals, Augst, Switzerland) and MRP14 (S36.48, BMA Biomedicals). For the detection of cytokines, adhesion molecules and growth factors we used anti-human TNFα (52B83; Monosan, Brussels, Belgium), anti-interleukin (IL)6 (Nephrology Department, Leiden University Medical Center, Leiden, The Netherlands), anti-IL10 (23738.111, R&D, Abingdon, UK), anti-IL18 (2d3b6, MD Biosciences, Minnesota, USA), anti-IL1β (2D8, ImmunoKontact, Oxford, UK), anti-intercellular adhesion molecule (ICAM)-1 (MEM111, Sanbio, Erembodegem, Belgium), anti-vascular cell adhesion molecule (VCAM) (1G11B1, Sanbio), anti-E-selectin (BBIG-E4, R&D), anti- vascular endothelial growth factor (VEGF) (N.010313 C-1, Santa Cruz, California, USA) and anti-basic fibroblast growth factor (bFGF) (F14220, BD). Staining of cellular markers was performed using a three-step immunoperoxidase method.^2^ For staining of cytokines, adhesion molecules and growth factors, biotinylated tyramine was used as amplification. For control sections the primary antibody was omitted or irrelevant immunoglobulins were applied.

### Digital image analysis

All sections were analysed at random by trained analysts blinded for clinical outcome. Images were acquired and analysed by computer-assisted image analysis using a Syndia algorithm on a Qwin-based analysis system (Leica, Cambridge, UK).^10^ A total of 18 high-power fields per marker were analysed. Cellular markers were expressed as positive cells/mm^2^ (counts/mm^2^). Staining of cytokines, adhesion molecules and growth factors was expressed as integrated optical density/mm^2 ^(IOD/mm^2^). CD68+ macrophages and TNFα expression were analysed separately in the intimal lining layer and the sublining.

### Statistical analysis

Independent Student t tests or Mann–Whitney U tests were used to detect significant differences in baseline parameters between responders and non-responders. The χ^2^ test was employed to compare the percentage of erosive, IgM-rheumatoid factor (RF) and anti-cyclic citrullinated peptide (CCP) positive patients in the responder and non-responder groups. Variables with a p value <0.1 in the univariable analysis were selected as possible predictors in a stepwise backward multivariable logistic regression model. Several exchangeable prediction sets were constructed based on the correlation structure among the potential predictors with strongly correlating predictors in different sets. The model with the highest explained variance according to Nagelkerke is reported. Correlations were assessed with the Pearson product-moment or Spearman rank-order correlation coefficients, whichever was appropriate. SPSS V.11.1.4 (Chicago, Illinois, USA) was used. Because the primary objective of this study was to find a pathogenetic mechanism in synovial tissue relating to clinical response, a per-protocol analysis was performed.

## RESULTS

### Enrolment, discontinuation and exclusion

A total of 143 patients were enrolled. The population was predominantly female (74%), RF positive (71%) with a mean (SD) age of 55 (13) years and mean disease duration of 125 (108) months. Erosions were present in 110 (77%) patients. The mean DAS28 score was 5.9 (1.0) at baseline and the mean MTX dose was 18.2 (8.5) mg/week. In three patients, a flare of arthritis at week 6 (n = 1) and week 12 (n = 2) was the reason for discontinuation. These patients were analysed as non-responders. Patients (n = 18) who violated the protocol or discontinued infliximab therapy (for reasons other than non-response) were excluded.

### Synovial tissue exclusion

Following strict quality control, 23 synovial biopsies were excluded by a blinded assessor for absence of an intimal lining layer. After excluding 18 patients from the response analysis and 23 synovial biopsies from the tissue analysis a total of 103 completely evaluable patients remained for the analysis of synovial tissue in combination with clinical response.

### Baseline patient characteristics

The remaining group of 103 patients who were analysed for response in combination with synovial tissue consisted of 71 females and 32 males, of whom 75% were rheumatoid factor positive. The mean (SD) age was 55 (13) years and the mean disease duration was 125 (110) months. Erosions were present in 79 (77%) patients. The mean DAS28 score was 5.9 (1.1) at baseline and the mean MTX dose was 18.8 (8.5) mg/week. Corticosteroids were used by 28 (27%) patients with a mean dose of 8.0 (2.9) mg/day. On average, patients had failed treatment with 2.2 DMARDs before inclusion in the study. No notable changes occurred in the baseline characteristics of the analysed 103 patients compared to the baseline characteristics of all 143 enrolled patients (see table 1).

**Table 1:** Baseline patient characteristics

	Analysed patients (n = 103)	Responders (n = 70)	Non-responders (n = 33)	p Value
Demographics:				
Age (years)	55 (13)	54 (13)	56 (12)	0.40
Female (%)	71 (69)	51 (73)	20 (61)	0.21
Disease status:				
Disease duration (months)	125 (110)	123 (111)	130 (110)	0.80
Erosive disease (%)	79 (77)	57 (81)	22 (67)	0.10
RF positive (%)	77 (75)	57 (81)	20 (61)	0.02
Anti-CCP positive (%)	78 (76)	57 (81)	13 (39)	0.08
DAS28	5.9 (1.1)	6.0 (1.0)	5.6 (1.2)	0.07
Patients global score (0–100 mm)	60 (22)	62 (21)	56 (23)	0.23
ESR (mm/h)	34 (24)	36 (23)	29 (25)	0.16
CRP (mg/dl)	22 (28)	24 (26)	19 (30)	0.36
Drug treatments:				
Previous DMARDs	2.2 (1.5)	2.1 (1.4)	2.4 (1.6)	0.42
Methotrexate (mg/week)	18.8 (8.5)	19.5 (8.2)	17.1 (8.9)	0.18
Receiving corticosteroids (%)	28 (27)	22 (31)	6 (18)	0.22
Receiving NSAIDs (%)	52 (50)	35 (50)	17 (52)	0.89

Mean (SD), median and interquartile range (IQR) or percentages are shown. p Values <0.05 (two-sided) were considered significant.

CCP, cyclic citrullinated peptide; CRP, C-reactive protein; DAS28, 28-joint Disease Activity Score; DMARD, disease-modifying antirheumatic drug; ESR, erythrocyte sedimentation rate; NSAID, non-steroidal anti-inflammatory drug; RF, rheumatoid factor.

### Clinical response

Of 103 patients, 70 (68%) were DAS28 responders and 33 (32%) non-responders at week 16. The clinical efficacy matched the expected 60–70% responders observed in previous randomised controlled trials with infliximab in patients with RA. The mean change in DAS28 was 1.84 (1.26) at week 16. For non-responders the average ΔDAS28 was 0.45 (0.58) compared to 2.52 (0.89) for responders. This difference was highly significant (p<0.001).

All baseline patient characteristics were also tested for differences between responders and non-responders. The DAS28 at baseline was nearly significant and was included in the prediction model (p = 0.07). The same holds for anti-CCP positivity (p = 0.08). Finally, there were significantly more rheumatoid factor positive patients in the responder compared to the non-responder group (p = 0.020), (see table 1).

### Baseline synovial TNFα expression and the number of major TNFα producing cells are associated with clinical response (ΔDAS28 ⩾1.2) after 16 weeks of TNFα blocking therapy

**Figure 1:**
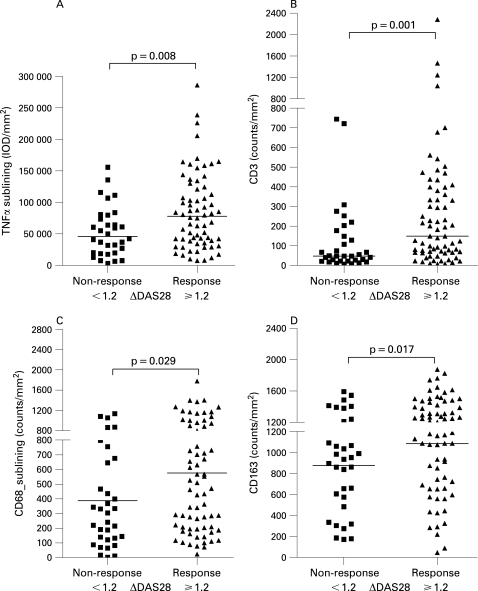
A. The median synovial sublining tumour necrosis factor (TNF)α expression was higher in responders compared to non-responders (p = 0.008). B. The median number of CD3+ T cells in responders vs non-responders (p = 0.001). (C) The mean number of CD68+ sublining macrophages was also higher in responders 576 (428), than in non-responders 387 (338) (p = 0.029). (D) CD163+ macrophages in responders vs non-responders (p = 0.017).

Scatter diagrams of the synovial predictors in individual patients in both response groups are presented in fig 1. TNFα expression levels were higher in the synovial sublining of responders compared to non-responders (p = 0.008) (fig 1A). Similarly, TNFα expression in the intimal lining layer was higher in responders compared to non-responders (p = 0.047). Table 2 shows the associations of synovial markers and response.

**Table 2:** Associations of studied synovial predictors with clinical response

	ΔDAS28 score		p Value
	Responders (n = 70) ⩾1.2	Non-responders (n = 33) <1.2	
Cytokines (IOD/mm^2^):			
TNFα lining	48 214 (29 116–88 971)	36 376 (23 394–54 271)	0.047
TNFα sublining	77 947 (38 710–123 535)	46 033 (19 890–78 129)	0.008
IL1β	56 278 (31 063–81 655)	54 484 (32 098–129 041)	0.814
IL6	50 537 (41 939)	38 364 (28 527)	0.135
IL10	131 106 (87 359)	115 964 (83 953)	0.415
IL18	9530 (12 086)	7009 (7470)	0.289
Cellular markers (counts/mm^2 ^)			
CD55	652 (418)	694 (389)	0.895
CD3	149 (66–385)	47 (22–163)	0.001
CD68 lining	374 (253)	295 (184)	0.130
CD68 sublining	576 (428)	387 (338)	0.029
CD163	1100 (432)	878 (433)	0.017
CD22	54 (99)	47 (111)	0.756
CD38	284 (384)	325 (556)	0.661
MRP8	139 (35–294)	53 (19–135)	0.018
MRP14	159 (34–526)	51 (18–166)	0.024
Adhesion molecules (IOD/mm^2^)			
ICAM	30 790 (33 016)	22 321 (21 706)	0.195
VCAM	80 065 (52 630)	70 701 (44 574)	0.387
E-Selectin	37 651 (40 134)	30 488 (29 972)	0.373
Growth factors (IOD/mm^2 ^)			
VEGF	18 252 (78 689)	5776 (5372)	0.374
bFGF	209 (736)	64 (163)	0.276

*Data are presented as mean (SD) or median (interquartile range), whichever appropriate. p Values<0.05 (two-sided) were considered significant.

bFGF, basic fibroblast growth factor; ICAM, intercellular adhesion molecule; IF, interferon; IOD, integrated optical density; MRP, myeloid related protein; TNF, tumour necrosis factor; VCAM, vascular cell adhesion molecule; VEGF, vascular endothelial growth factor.

Because macrophages are known to be the main TNFα producing cells in the synovium, the number of macrophages was studied. The mean number of CD68+ sublining macrophages was significantly higher in responders than in non-responders (p = 0.029) (fig 1C). Similarly, the number of CD163+ resident tissue macrophages as well as the number of infiltrating MRP8+ and MRP14+ macrophages was higher in responders (p = 0.017, p = 0.018 and p = 0.024, respectively) (fig 1D). Finally, the median number of CD3+ T cells was higher in responders (p = 0.001) (fig 1B).

### Baseline TNFα is the only significant synovial predictor of response

The relationship between TNFα expression and clinical response was confirmed by stepwise backward multivariable logistic regression analysis. Due to multicolinearity among predictors, 10 exchangeable bivariate prediction sets were constructed consisting of (1) TNFα expression in either the intimal lining layer or synovial sublining with (2) one out of five cellular markers. This revealed that TNFα expression in the synovial sublining was the only independent, early determinant of therapy response (p = 0.011), explaining just about 10% of the variance in response to therapy (R^2^ = 0.099, according to Nagelkerke, odds ratio (OR) = 1.013). Likewise, TNFα expression in the intimal lining layer rather than synovial sublining explained 9% of the variance in response to therapy (p = 0.020), (R^2^ = 0.089, OR = 1.017). Thus, after adjustment for TNFα in a bivariate logistic regression model including either the numbers of CD68+ sublining macrophages, CD163+, MPR8+, MRP14+ macrophages, or CD3+ T cells, these cellular markers were no longer significantly associated with response. The removal of these cellular markers from the model is consistent with the notion that these cells are the main producers of TNFα.

### Contribution of disease activity at baseline to the prediction of response

To assess the value of a combined prediction model consisting of clinical and synovial data we added the relevant clinical variables (DAS28 score, the presence of IgM-RF or anti-CCP antibodies) to the 10 bivariate synovial models. With stepwise backward multivariate logistic regression this resulted in a significant prediction model with TNFα expression in the synovial sublining (p = 0.008, OR = 1.014) and the DAS28 (p = 0.031, OR = 1.611) with an increased explained variance of 17% (R^2^ = 0.172). A similar analysis except with TNFα expression in the intimal lining layer (p = 0.023, OR = 1.017) resulted in a significant model including the DAS28 (p = 0.044, OR = 1.588) and the presence of anti-CCP antibodies (p = 0.046, OR = 3.038) (R^2^ = 0.182).

### Baseline IL1β, IL6, IL18 and IL10 expression is not associated with response to TNFα blocking therapy

In addition to TNFα we studied the expression of other cytokines such as IL18, which is believed to operate mainly upstream of TNFα, while IL1β and IL6 appear to act mainly downstream of TNFα. Despite the known interplay of these cytokines with TNFα we found no relationship between synovial expression levels of either of these cytokines and clinical response. We did find, however, a significant correlation between the level of IL1β expression and TNFα expression (r = 0.306, p = 0.014). The expression level of IL10 was not different between response groups (see table 2).

### Baseline expression of adhesion molecules and angiogenic factors is not associated with response to TNFα blocking therapy

In accordance with their function in leukocyte recruitment a significant correlation was found between the number of sublining macrophages on the one hand and the expression of the vascular adhesion molecules E-selectin (Spearman rho correlation coefficient 0.275–0.536, p<0.01) on the other. However, the level at which the adhesion molecules were expressed in the synovium at baseline did not differ between responders and non-responders. Furthermore, VEGF is known to mediate angiogenesis and is regulated by pro-inflammatory cytokines such as TNFα. However, no association was found between VEGF expression and response (table 2). Similarly, no difference was found in the expression of bFGF.

## DISCUSSION

The primary objective of this study was to investigate whether immunohistological assessment of the cell infiltrate and cytokine expression in the synovium prior to initiation of TNFα blocking therapy could predict clinical response in patients with RA. We could confirm our hypothesis that the level of synovial TNFα expression is a significant early predictor of the response. This was shown by increased TNFα expression levels in the intimal lining layer and synovial sublining of responding compared to non-responding patients. In line with these findings, there was increased infiltration by macrophages, including CD163+ resident tissue macrophages and MRP8+ and MRP14+ infiltrating macrophages, as well as T cells in responders vs non-responders. It is important to note that these cells are the main source of TNFα in the synovium of patients with RA.^11^

Consistent with the clinical experience that the response to TNFα blockade is not a dichotomous phenomenon,^12^ there was no distinct threshold value in TNFα expression in the synovium of patients with RA. Multivariate logistic regression analysis of synovial markers showed that TNFα expression in the sublining could explain about 10% of the variance in response to therapy. After adjusting for disease activity at baseline this further increased to 17%. Hence, the predictive value of synovial TNF expression is statistically significant, but overall limited. This clearly indicates that variables other than synovial TNFα expression are involved as well. The results presented here show for the first time that the expression of IL1β, IL6, IL18, IL10, E-selectin, ICAM-1, VCAM-1, VEGF and bFGF is not associated with clinical response to anti-TNFα treatment. Thus, the features of synovial inflammation at baseline in responders to anti-TNF therapy compared to non-responders do not merely represent a greater amount of inflammation, but the results presented here underscore the importance of specific inflammatory pathways. Future work should expand the search for other biomarkers and molecular networks to better understand the variable response to anti-TNFα therapy.

The search for predictors of response is important in the context of personalised medicine, which may be an effective approach to increase the percentage of patients exhibiting a robust response to a given treatment. Previous work has clearly suggested that RA consists of different pathogenetic subsets, leading to common signs and symptoms associated with what we at present define clinically as RA.^2^ ^3^ ^13^ Thus, it is conceivable that, for instance, TNFα expression is more important in some patients than in others, and that TNF blockade could be more effective in the former. Although we are as yet not able to predict the response sufficiently to select patients who are likely to have a beneficial response to a specific treatment before initiation of therapy and to guide treatment decisions, the results of the present study do provide proof of principle that this might be achieved by further optimisation of the biomarkers or perhaps combinations with other clinical and biological variables that need to be identified. The relevance of this approach is underscored by the expanding array of biological therapies and their costs.^14^

There is no evidence that simple measurement of plasma TNFα levels can be used to predict clinical response to TNFα blockade. As it appears logical that patients producing high levels of TNFα at the site of inflammation are more likely to benefit from TNFα blockade than those with lower TNFα levels, we focused on the synovium as the primary target of RA. The association between pretreatment TNFα expression in the synovium and clinical response to 3 mg/kg of infliximab described here in 103 patients with RA confirms and extends a trend observed in a pilot study in patients with RA where 4 out of 8 patients who met the ACR50 response criteria at 2 weeks after initiation of infliximab (10 mg/kg) therapy were those with the highest levels of TNFα expression in the synovium, as shown by immunohistochemistry.^7^ Of interest, the findings are also in accordance with a recent study suggesting that clinical response at 52 weeks is associated with significantly higher circulating TNFα bioactivity (measured by an in vitro bioassay) at baseline in responding compared to non-responding patients.^6^ Together, these studies support the notion that the initial clinical response to TNFα blockade is related to pretreatment levels of TNFα production. In patients who initially respond, but loose response over time, other mechanisms such as formation of antibodies against the drug may be operative.^15^

In conclusion, the results presented here show proof of principle that the heterogeneous response to TNFα blockade is associated with TNFα expression in the inflamed synovium. Future work should expand the search for other biomarkers and molecular networks as well as combinations with clinical variables. Thus, the prediction of how a patient will respond might come in reach of the treating doctor.
